# Ras-associated autoimmune leukoproliferative disorder associated with immune thrombotic thrombocytopenic purpura

**DOI:** 10.1016/j.rpth.2026.106933

**Published:** 2026-09-23

**Authors:** Matthew A. Carter, Andrew J. Innes, Monica Sen, Elisabet Nadal-Melsio, Asad Luqmani, Jane F. Apperley, Marie Scully

**Affiliations:** 1 Haemostasis Research Unit, Institute of Cardiovascular Science, University College London, London, UK; 2 Department of Haematology, University College London Hospitals, London, UK; 3 Department of Haematology, Imperial College Healthcare NHS Trust, Hammersmith Hospital, London, UK; 4 Department of Immunology and Inflammation, Centre for Haematology, Imperial College London, UK

**Keywords:** RALD, Ras-associated autoimmune leukoproliferative disorder, thrombotic thrombocytopenic purpura, TTP

## Abstract

**Background:**

Ras-associated autoimmune leukoproliferative disorder (RALD) is a rare, nonmalignant condition characterized by monocytosis, lymphoproliferation, and autoimmunity, driven by *KRAS* or *NRAS* variants. Immune thrombotic thrombocytopenic purpura (iTTP) results from autoantibodies against ADAMTS13 (a disintegrin and metalloproteinase with a thrombospondin type 1 motif, member 13).

**Key Clinical Question:**

Can RALD co-occur with iTTP, and how can it be diagnosed?

**Clinical Approach:**

A patient presenting with neutropenia, monocytosis, and thrombocytopenia was initially diagnosed with chronic myelomonocytic leukemia. She later developed splenomegaly, lymphadenopathy, and iTTP. Reevaluation identified a somatic *KRAS* G12A variant in peripheral blood, prompting a diagnosis of RALD. Flow cytometry demonstrated immunophenotypic changes involving B cells, T cells, and monocytes. The patient had four ADAMTS13 relapses, responsive to rituximab.

**Conclusion:**

This case highlights co-occurrence of RALD and iTTP, extending the spectrum of Ras-associated autoimmune cytopenias.

## Essentials

• RALD is a rare autoimmune condition.
• We describe the co-occurrence of RALD with iTTP.
• RALD can be mistaken for several other blood conditions, for which treatment is different.
• This extends the range of other autoimmune conditions that have been associated with RALD.

## Case Presentation

A 44-year-old African woman was referred in 2010 after neutropenia and thrombocytopenia were identified preoperatively. Initial blood tests showed mild anemia (hemoglobin, 116 g/L), white cell count (WCC) of 3.5 × 10^9^/L (monocytes, 1.8 × 10^9^/L; lymphocytes, 1.2 × 10^9^/L; and neutrophils, 0.4 × 10^9^/L), and platelet count of 36 × 10^9^/L (Figure 1A). Imaging revealed mild splenomegaly (15.5 cm on computed tomography), and a bone marrow sample showed 40% monocytes and a small lymphocytic infiltrate. Tests for a myeloproliferative neoplasm, including JAK2 V617F and BCR-ABL, were negative. A working diagnosis of chronic myelomonocytic leukaemia (CMML) was made.

**Figure 1:**
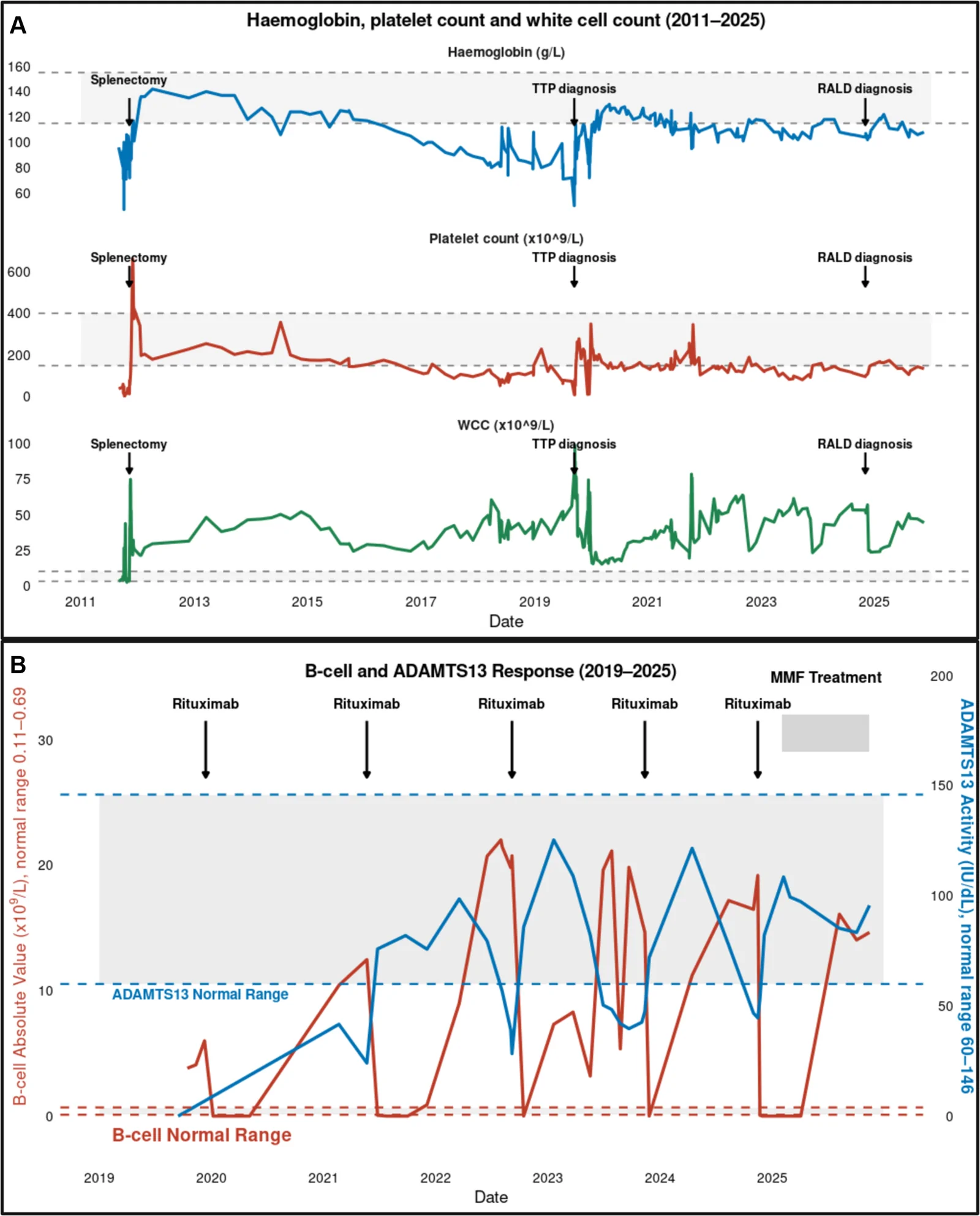
Full blood count, B-cell, and ADAMTS13 (a disintegrin and metalloproteinase with a thrombospondin type 1 motif, member 13) monitoring. (A) Full blood count parameters from initial assessment to present, highlighting the changes seen postsplenectomy, immune thrombotic thrombocytopenic purpura (iTTP), and Ras-associated autoimmune leukoproliferative disorder (RALD) diagnosis. (B) B-cell count (red) and ADAMTS13 activity (blue) in relation to treatment with rituximab (arrows) over the period 2019-2025. The normal range of B cells is represented by the dashed red lines, and the normal range of ADAMTS13 activity is represented by the dashed blue lines. Rituximab courses were delivered as 2 to 4 divided doses, with the arrows representing the date of the first dose of that course. Mycophenolate mofetil (MMF) treatment is represented by the gray bar and continues at present.

On repeat bone marrow sampling 18 months later, monocytosis was reconfirmed, with CD68^+^ cells accounting for 15% to 20% of cells and no evidence of B-cell or T-cell clonality. Concurrently, a groin mass biopsy showed a lymph node with reactive changes, a monocytic infiltrate, and no evidence of lymphoma. These findings were atypical but considered most consistent with CMML. She was treated with red cell transfusion support and actively monitored without disease-modifying therapy.

### Worsening splenomegaly, splenectomy, and persistent lymphadenopathy

Increasing splenomegaly (29.5 cm) was thought to contribute to her cytopenias (hemoglobin, 84 g/L; WCC, 6.4 × 10^9^/L; and platelet count, 35 × 10^9^/L), and splenectomy was planned. She underwent splenectomy in late 2011, complicated by perioperative sepsis requiring intensive care. Histopathology showed necrotic foci with areas of proliferation and excess monocytes, felt to be most in keeping with CMML. Postsplenectomy, the platelet count normalized, but a persistent mixed leukocytosis developed (WCC, 30-60 × 10^9^/L). Abdominal lymphadenopathy was noted in 2014 but was not amenable to biopsy. In 2017, skin lesions showed lichenoid inflammation and mild dermal lymphohistiocytic infiltrate, attributed to CMML and resolving with topical steroid treatment. A positron emission tomography/computed tomography scan in 2018 confirmed progression of the lymphadenopathy, and a biopsy again demonstrated reactive features. Molecular testing of peripheral blood in 2018 revealed a somatic *KRAS* NM_033360.3 c.35G>C p.(Gly12Ala) variant with a variant allele frequency (VAF) of 48%, without other myeloid-associated variants; however, the significance of this result was not yet clear.

### Diagnosis of iTTP

Nearly 10 years from the initial presentation, the patient developed acute thrombocytopenia in September 2019 (platelet count, 12 × 10^9^/L) (Figure 1A), severe anemia (hemoglobin, 50 g/L), signs of microangiopathic hemolytic anemia including schistocytes on a peripheral blood film together with a marked leukocytosis (WCC, 80.9 × 10^9^/L; neutrophils, 37.2 × 10^9^/L; lymphocytes, 19 × 10^9^/L; and monocytes, 23.7 × 10^9^/L). Thrombotic thrombocytopenic purpura (TTP) was suspected, and plasma exchange was initiated. ADAMTS13 (a disintegrin and metalloproteinase with a thrombospondin type 1 motif, member 13) activity was <5 IU/dL (60-146 IU/dL), with presence of anti–ADAMTS13 antibody, confirming immune TTP (iTTP). Plasma exchange and corticosteroids led to an initial response, with an improvement in platelet count and a reduction in WCC to 40.2 × 10^9^/L. She had an early relapse of iTTP in December 2019, at which point, the WCC was 52.3 × 10^9^/L and the CD19 cell count, a B-cell marker, was 6.0 × 10^9^/L (0.11-0.69 × 10^9^/L). She was treated with further plasma exchanges, pulsed methylprednisolone (1 g × 3), and rituximab (4 doses of 375 mg/m^2^ weekly for 4 weeks).

Following treatment, the lymphocyte count normalized but monocytosis persisted at a lower level. Antinuclear, anti-Ro, and anti-La autoantibody screens were persistently positive. Over the subsequent 5 years, a rising leukocytosis was associated with a falling ADAMTS13 activity level on 4 occasions. The CD19 count in peripheral blood increased markedly between courses of rituximab, peaking at 21.1 × 10^9^/L or 85.4% of lymphocytes (4.16%-26.1%) (Figure 1B). Immunoglobulins were elevated; IgG was at 18 to 21 g/L (7-16 g/L) and IgA at 6.0 g/L (0.7-4 g/L), with no paraprotein detected.

### Reassessment and diagnosis of RALD

The diagnosis of CMML, however, remained uncertain as repeated flow cytometry on peripheral blood showed monocyte partitioning was normal, and there was a lack of progression over many years. Molecular testing confirmed no lymphoid clonality with repeatedly negative immunoglobulin gene rearrangement and negative T-cell clonality. Next-generation sequencing testing of peripheral blood in 2025 reconfirmed the c.35G>C p.(Gly12Ala) *KRAS* variant, previously identified in RALD and other neoplasms [1]. Paired analysis of whole blood and sorted CD3^+^ T cells demonstrated the variant in both compartments (VAF, 47% and 8%, respectively), confirming multilineage involvement; the differing variant frequencies were consistent with a somatic rather than a germline variant, as a germline variant would be expected to have a similar VAF between cell types.

On review of her sustained monocytosis, splenomegaly, and lymphoproliferation and presentation with another autoimmune disease (iTTP), a diagnosis of Ras-associated autoimmune leukoproliferative disorder (RALD) was considered most likely [2]. In addition to elective rituximab, required for repeated ADAMTS13 relapses, mycophenolate mofetil was introduced as an additional immunosuppressive agent. Since its introduction, the patient has had a significant improvement in her symptoms and quality of life.

## Results and Discussion

Peripheral blood immunophenotyping, performed in iTTP remission, demonstrated a profile compatible with RALD rather than CMML or iTTP alone (Figure 2, Supplementary Table 1, and Supplementary Figure 1). There was a reduction in circulating CD3^+^ T lymphocytes (Figure 2A) without expansion of CD3^+^TCRαβ^+^CD4^−^CD8^−^ double-negative T cells (Figure 2C), differentiating the condition from autoimmune lymphoproliferative syndrome (ALPS), where an increase in double-negative T cells would be expected [1]. In contrast, there was a marked polyclonal B-cell expansion with increased CD10 expression (Figure 2D) and a predominance of CD16^+^ monocytes (Figure 2E), a pattern previously described in RALD and not in keeping with the classical monocyte expansion seen in CMML. These findings supported the diagnosis of RALD in the context of iTTP [1,3].

**Figure 2:**
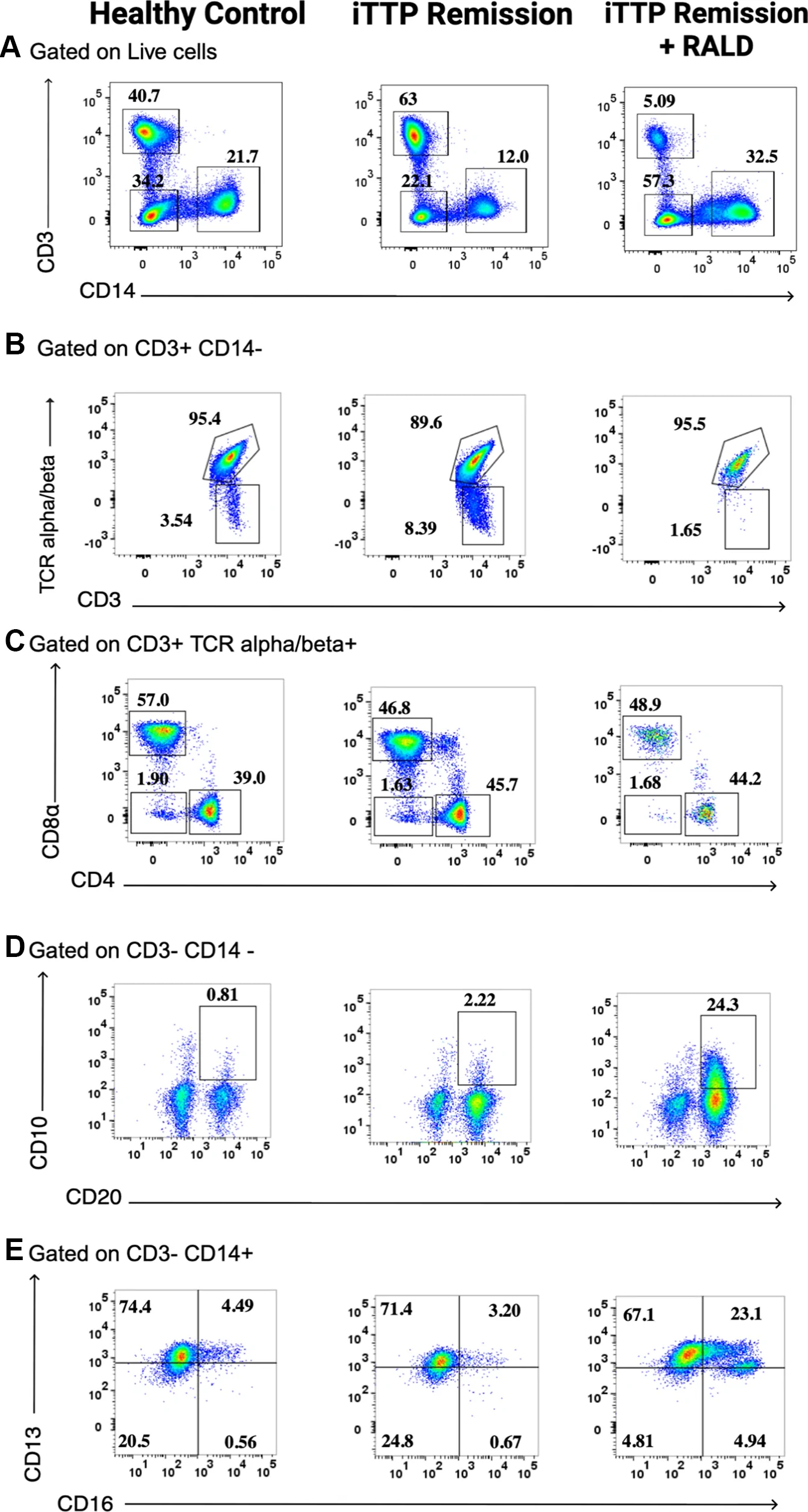
Flow cytometry plots of peripheral blood mononuclear cells (PBMCs) from a healthy control, patient with immune thrombotic thrombocytopenic purpura (iTTP) remission, and patient with Ras-associated autoimmune leukoproliferative disorder (RALD) and iTTP. These panels show immune cell populations gated on live cells and relevant subpopulations to demonstrate changes in T-cell, B-cell, and monocyte compartments. The numbers on plots represent percentages of cells within a gated region. (A) Reduction in CD3^+^CD14^−^ T lymphocytes in RALD. (B) Proportion of CD3^+^TCRαβ^+^ T cells. (C) Identification of CD4^−^CD8^−^ (double-negative) T cells. (D). Increased CD10 expression in CD20^+^ cells in the patient with RALD. (E) CD14^+^ monocytes show an increase in CD16 expression compared with healthy and iTTP control samples, which contrasts with the increase in CD16^−^ classical monocytes seen in chronic myelomonocytic leukemia. TCR, T-cell receptor.

Both RALD and iTTP are rare autoimmune diseases, and we present, to our knowledge, the first case of the 2 rare conditions co-occurring in an adult, an association only previously described in a child [4]. RALD is a chronic, nonmalignant autoimmune condition, characterized by monocytosis, lymphoproliferation, splenomegaly, and autoimmune phenomena [5]. A subgroup of patients with ALPS and somatic RAS mutations was described in 2007 [6], and this led to the description of RALD and its consideration as a separate entity in 2011 due to different underlying genetic changes and a non–Fas-mediated defect in apoptosis (Table) [[10], [7], [8], [9]]. iTTP is a microangiopathic hemolytic anemia caused by autoantibody-mediated deficiency of the ADAMTS13 protease, with an incidence of 2 to 6 per million per year [11,12]. ADAMTS13 deficiency leads to platelet-rich microthrombi, causing organ damage and failure; if left untreated, mortality rate exceeds 90% [13,14].

**Table:** A comparison of the genetic changes, clinical features, biomarkers, and management strategies in RALD, ALPS, and CMML

Parameter	RALD	ALPS	CMML
Genetic driver(s)	Somatic (acquired) activating *KRAS* or *NRAS* variants, in the hematopoietic lineage [1]	Germline or somatic defects in *FAS* most common, less often *FASL*, *CASP10*, or other related genes [7]	Neoplastic clonal hematopoiesis, mutations commonly seen in *TET2*, *SRSF2*, *ASXL1*, *KRAS*, and *NRAS* [8]
Age of presentation	Childhood or adulthood	Typically childhood	Predominantly older adults
Clinical features	Splenomegaly, variable lymphadenopathy, and autoimmune cytopenias	Splenomegaly, chronic nonmalignant lymphadenopathy, hepatomegaly, and autoimmune cytopenias	Systemic symptoms with splenomegaly, cytopenias, and complications of bone marrow failure
Monocytosis	Yes (characteristic and persistent)	No	Yes (diagnostic requirement; sustained (>3 mo) ≥0.5 × 10^9^/L; and monocytes > 10% of leukocytes)
Autoimmune cytopenias	Reported	Common (up to one-third of patients)	Reported but uncommon; cytopenias usually reflect dysplasia or bone marrow failure
Apoptosis defect	RAS-driven signaling defect, not mediated by Fas pathway	Defect in Fas-mediated apoptosis	No consistent apoptosis defect described
Double-negative T cells (CD4^−^/CD8^−^TCRαβ^+^)	Normal or only slightly increased	Markedly increased (diagnostic hallmark)	Not characteristic
ALPS biomarkers (sFASL and IL-10)	Usually normal	Typically elevated	Not characteristic
Malignancy risk	No established progression and monitoring required	Increased lymphoma risk	Risk of transformation to AML
Management focus	Monitoring, treatment of autoimmune cytopenias, and avoiding misdiagnosis as CMML or JMML	Genetic counseling, lymphoma surveillance, treatment of autoimmune cytopenias, and immunosuppression [9]	Risk-adapted therapy: monitoring, cytoreduction, hypomethylating agents, and allogeneic transplant in selected patients

ALPS, autoimmune lymphoproliferative syndrome; AML, acute myeloid leukaemia; CMML, chronic myelomonocytic leukemia; IL, interleukin; JMML, juvenile myelomonocytic leukaemia; MDS, myelodysplastic syndrome; MPN, myeloproliferative neoplasm; RALD, Ras-associated autoimmune leukoproliferative disorder; sFASL, soluble Fas ligand.

RALD has features that overlap with CMML, including somatic *KRAS* or *NRAS* mutations in peripheral blood and monocytosis; however, it reflects nonmalignant immune dysregulation rather than a clonal myeloid neoplasm [2]. Diagnosis may be delayed due to lack of awareness, overlapping features with other conditions (Table), rarity, and the lack of universally accepted diagnostic criteria. In this patient, there was a lack of other myeloid variants apart from the *KRAS* variant, an atypical clinical course, and significantly raised CD19 count and IgG levels, which would not be expected in CMML (Box 1). Other causes of chronic monocytosis with immune dysregulation were also considered less likely: germline RASopathies were not supported by the adult-onset presentation and somatic variant, clonal hematopoiesis with inflammatory manifestations did not fit with the molecular or clinical findings, and other inflammatory disorders could not account for the persistent monocytosis with a somatic *KRAS* variant.

Box 1 **Clinical and laboratory features suggestive of RALD in a patient with iTTP**

• Persistent monocytosis, with flow cytometry findings atypical for CMML.
• Lymphoproliferation or splenomegaly without evidence of malignancy.
• Persistent cytopenias without other identified causes.
• Somatic activating *KRAS* or *NRAS* variants in peripheral blood.

RALD is very rare (estimated incidence < 1 per million), with the literature comprising mostly case reports or series [15]. Management focuses on monitoring for autoimmune complications and treating these. In contrast to ALPS, RALD has no Fas-pathway defect, with normal ALPS-associated markers (interleukin 10 and soluble Fas ligand); in this patient, functional studies of Ras pathway activation, Fas-mediated apoptosis, and these biomarkers were not performed, and the diagnosis rested on the combined clinical, molecular, and immunophenotypic findings. In RALD, it has been suggested that constitutive activation of Ras may overcome B-cell tolerance and impair the function of the proapoptotic protein BIM (BCL2-interacting mediator of cell death), preventing removal of autoreactive B cells and leading to the development of autoimmunity [16]. Autoimmune complications including autoimmune hemolytic anaemia, immune thrombocytopenia, inflammatory arthritis, and systemic lupus erythematosus are described [2,[17], [18], [19]].

This case illustrates that RALD may masquerade as a myeloid malignancy for which the treatment pathway is significantly different. In retrospect, the patient’s earlier thrombocytopenia responsive to splenectomy may have been either immune thrombocytopenia or TTP; however, this cannot be confirmed as ADAMTS13 testing was not performed. In patients with suspected myeloproliferative diseases, negative myeloproliferative neoplasm testing, and relevant clinical features, testing for somatic Ras mutations should be considered to identify RALD. Repeated rituximab treatment for ADAMTS13 relapses was associated with a reduction in leukocyte count, CD19, and IgG along with improvement of the ADAMTS13, and the addition of a steroid sparing agent (mycophenolate) was associated with a significant improvement in the patient’s health and well-being.

Activating Ras mutations have been proposed to disrupt B-cell tolerance and permit survival of autoreactive B cells, and this mechanism is of theoretical interest in iTTP, where pathogenic anti–ADAMTS13 antibodies drive the disease. However, we emphasize that this case provides no direct evidence linking Ras activation to anti–ADAMTS13 autoimmunity and that the mechanism underlying anti–ADAMTS13 autoimmunity remains unknown. Our findings establish a clinical association between RALD and iTTP; whether Ras-mediated immune dysregulation contributed to the pathogenesis of iTTP, or the 2 conditions co-occurred by chance, cannot be determined from a single case and remains a hypothesis for future studies.

## Figures and Tables

**Supplementary Figure S1:**
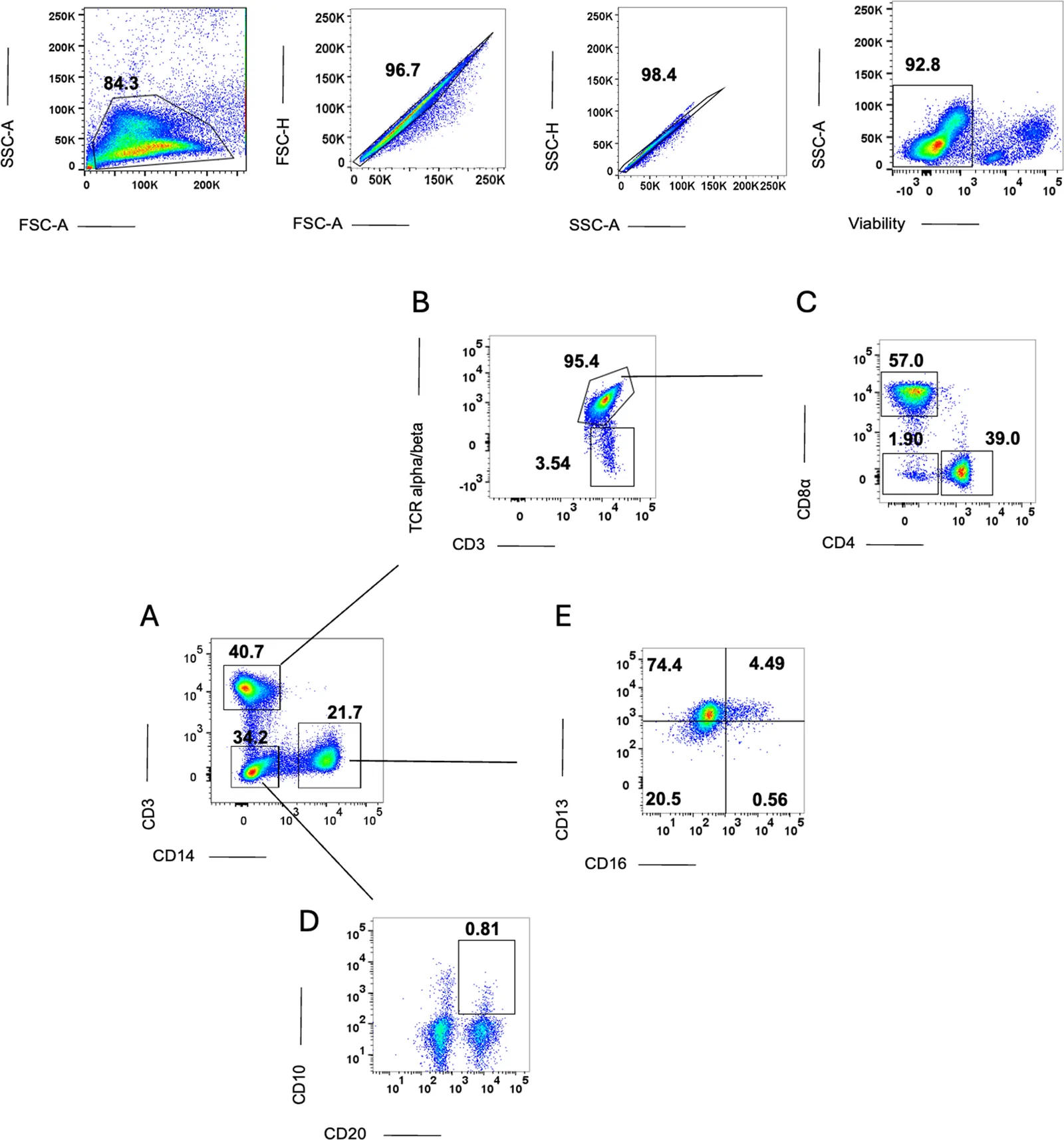


## References

[1] Calvo K.R., Price S., Braylan R.C., Oliveira J.B., Lenardo M., Fleisher T.A. (2015). JMML and RALD (Ras-associated autoimmune leukoproliferative disorder): common genetic etiology yet clinically distinct entities. Blood.

[2] Neven Q., Boulanger C., Bruwier A., de Ville de Goyet M., Meyts I., Moens L. (2021). Clinical spectrum of Ras-associated autoimmune leukoproliferative disorder (RALD). J Clin Immunol.

[3] Blanchard-Rohner G., Ragotte R.J., Junker A.K., Sharma M., Del Bel K.L., Lu H.Y. (2021). Idiopathic splenomegaly in childhood and the spectrum of RAS-associated lymphoproliferative disease: a case report. BMC Pediatr.

[4] Ziv A., Dardik R., Yacobovich J., Uziel Y., Haviv R., Avishai E. (2024). Atypical presentations of pediatric-acquired thrombotic thrombocytopenic purpura. J Pediatr Hematol Oncol.

[5] Rivalta B., Attardi E., Cifaldi C., Rosti V., Pacillo L., Hajrullaj H. (2024). Natural history of Ras-associated autoimmune leukoproliferative disorder: a 20-year follow-up of a NRAS-mutated patient excluding a malignant progression. Br J Haematol.

[6] Oliveira J.B., Bidère N., Niemela J.E., Zheng L., Sakai K., Nix C.P. (2007). NRAS mutation causes a human autoimmune lymphoproliferative syndrome. Proc Natl Acad Sci U S A.

[7] Oliveira J.B., Bleesing J.J., Dianzani U., Fleisher T.A., Jaffe E.S., Lenardo M.J. (2010). Revised diagnostic criteria and classification for the autoimmune lymphoproliferative syndrome (ALPS): report from the 2009 NIH International Workshop. Blood.

[8] Patnaik M.M., Tefferi A. (2024). Chronic myelomonocytic leukemia: 2024 update on diagnosis, risk stratification and management. Am J Hematol.

[9] Rao V.K., Oliveira J.B. (2011). How I treat autoimmune lymphoproliferative syndrome. Blood.

[10] Niemela J.E., Lu L., Fleisher T.A., Davis J., Caminha I., Natter M. (2011). Somatic KRAS mutations associated with a human nonmalignant syndrome of autoimmunity and abnormal leukocyte homeostasis. Blood.

[11] Scully M., Rayment R., Clark A., Westwood J.P., Cranfield T., Gooding R. (2023). A British Society for Haematology Guideline: diagnosis and management of thrombotic thrombocytopenic purpura and thrombotic microangiopathies. Br J Haematol.

[12] Scully M., Yarranton H., Liesner R., Cavenagh J., Hunt B., Benjamin S. (2008). Regional UK TTP Registry: correlation with laboratory ADAMTS 13 analysis and clinical features. Br J Haematol.

[13] Furlan M., Robles R., Lämmle B. (1996). Partial purification and characterization of a protease from human plasma cleaving von Willebrand factor to fragments produced by in vivo proteolysis. Blood.

[14] Sadler J.E. (2008). Von Willebrand factor, ADAMTS13, and thrombotic thrombocytopenic purpura. Blood.

[15] He T.Y., Li C.R., Xia Y., Liang F.F., Luo Y., Yang J. (2017). [Ras-associated autoimmune leukoproliferative disorder: a report of 2 cases and literature review]. Zhonghua Er Ke Za Zhi Chin J Pediatr.

[16] Riller Q., Rieux-Laucat F. (2021). RASopathies: from germline mutations to somatic and multigenic diseases. Biomed J.

[17] Xie W., Fan G., Wiszniewska J., Press R.D., Yang F. (2022). Histopathological and molecular findings in a patient with Ras-associated autoimmune leukoproliferative disorder. Hum Pathol Rep.

[18] Wang W., Zhou Y., Zhong L., Wang L., Tang X., Ma M. (2019). RAS-associated autoimmune leukoproliferative disease (RALD) manifested with early-onset SLE-like syndrome: a case series of RALD in Chinese children. Pediatr Rheumatol Online J.

[19] Toyoda H., Deguchi T., Iwamoto S., Kihira K., Hori H., Komada Y. (2018). Weekly rituximab followed by monthly rituximab treatment for autoimmune disease associated with RAS-associated autoimmune leukoproliferative disease. J Pediatr Hematol Oncol.

