# Supplementary material for: Ras-associated autoimmune leukoproliferative disorder associated with immune thrombotic thrombocytopenic purpura
Source: Res Pract Thromb Haemost. 2026 Sep 23;10(6):106933. doi: 10.1016/j.rpth.2026.106933 (PMC13633909; doi:10.1016/j.rpth.2026.106933)
Supplement: Supplementary file 1 [file mmc1.docx]

**Supplementary Table 1.** Quantitative summary of peripheral blood mononuclear cell flow cytometry populations across the healthy control, iTTP remission control and RALD +iTTP patient. Values are the percentage within the stated parent gate, corresponding to the gated regions shown in Figure 2. Populations were defined using the sequential gating strategy shown in Supplementary Figure 1.

| Population (parent gate) | Panel (Figure 2) | Healthy control (%) | iTTP remission (%) | RALD + iTTP patient (%) |
| --- | --- | --- | --- | --- |
| CD3+ CD14- T lymphocytes (of live cells | A | 40.7 | 63.0 | 5.09 |
| CD3+ TCRαβ+ T cells (of CD3+ CD14-) | B | 95.4 | 89.6 | 95.5 |
| CD4- CD8- double-negative T cells (of CD3+ TCRαβ+) | C | 1.90 | 1.63 | 1.68 |
| CD10+ B cells (of CD3- CD14- CD20+) | D | 0.81 | 2.22 | 24.3 |
| CD16+ monocytes (CD13+ CD16+, of CD3- CD14+) | E | 4.49 | 3.20 | 23.1 |

**Supplementary Figure 1.** Sequential gating strategy for peripheral blood mononuclear cell (PBMC) immunophenotyping. Representative plots from the healthy control sample. PBMCs were identified on forward and side scatter (FSC-A/SSC-A) and doublets excluded on FSC-A/FSC-H and SSC-A/SSC-H, live cells were selected using a viability dye (top row). Live cells were then gated on CD3 vs CD14 (Panel A) to define three populations: CD3+ CD14- T lymphocytes, CD3- CD14- cells, and CD3- CD14+ monocytes. T lymphocytes were assessed for TCRαβ expression (Panel B) and CD3+ TCRαβ+ cells were then examined for CD4 and CD8 to identify CD4- CD8- double negative T Cells (Panel C). CD3- CD14- cells were assessed for CD10 and CD20 to identify CD10+ B cells (Panel D), and CD3- CD14+ monocytes were assessed for CD13 and CD16 to identify CD16+ monocytes (Panel E). Numbers on plots represent the percentage of cells within each gated region. Panel letters correspond to the panels of Figure 2, with the same gating strategy applied to all three samples shown in Figure 2.
